# Rates of Water Loss and Uptake in Recalcitrant Fruits of *Quercus* Species Are Determined by Pericarp Anatomy

**DOI:** 10.1371/journal.pone.0047368

**Published:** 2012-10-11

**Authors:** Ke Xia, Matthew I. Daws, Wolfgang Stuppy, Zhe-Kun Zhou, Hugh W. Pritchard

**Affiliations:** 1 Key Laboratory of Biodiversity and Biogeography, Kunming Institute of Botany, Chinese Academy of Sciences, Kunming, China; 2 Germplasm Bank of Wild Species, Kunming Institute of Botany, Chinese Academy of Sciences, Kunming, China; 3 Alcoa of Australia Ltd, Pinjarra, Western Australia, Australia; 4 Seed Conservation Department, Royal Botanic Gardens, Kew, United Kingdom; 5 Xishuangbanna Tropical Botanical Garden, Chinese Academy of Sciences, Mengla, Yunnan, China; University of Maryland, United States of America

## Abstract

Desiccation-sensitive recalcitrant seeds and fruits are killed by the loss of even moderate quantities of water. Consequently, minimizing the rate of water loss may be an important ecological factor and evolutionary driver by reducing the risk of mortality during post-dispersal dry-spells. For recalcitrant fruits of a range of *Quercus* species, prolonged drying times have been observed previously. However, the underlying mechanism(s) for this variation is unknown. Using nine *Quercus* species we investigated the major route(s) of water flow into and out of the fruits and analysed the relative importance of the different pericarp components and their anatomy on water uptake/loss. During imbibition (rehydration), the surface area of the cupule scar and the frequency and area of the vascular bundles contained therein were significantly correlated with the rates of water uptake across the scar. The vascular bundles serving the apex of the fruit were a minor contributor to overall water. Further, the rate of water uptake across the remainder of the pericarp surface was significantly correlated with the thickness of the vascularised inner layer in the pericarp. Fruits of *Q. franchetii* and *Q. schottkyana* dried most slowly and had a comparatively small scar surface area with few vascular bundles per unit area. These species inhabit drier regions than the other species studied, suggesting these anatomical features may have ecological value by reducing the risk of desiccation stress. However, this remains to be tested in the field.

## Introduction

Recalcitrant (desiccation-sensitive) fruits and seeds are dispersed with high water contents (typically >40% on a fresh weight basis) and are killed by drying to even comparatively high water contents (e.g. drying ≤20–30% fwt basis; [1]). Consequently, mechanisms that minimize the rate of water loss have the potential to be of ecological importance by reducing the risk of mortality during post dispersal dry spells. Thus, desiccation sensitivity has obvious disadvantages in the event of dry spells after dispersal. However, recent studies have suggested that producing recalcitrant seeds can also be advantageous: many recalcitrant seeds reduce the risk of desiccation and seed predation by germinating rapidly and therefore allocate proportionately more resources to the seed embryo (as opposed to defensive covering structures) than desiccation tolerant seeds and fruits [2], [3]. Whilst potentially important, seed morphological and anatomical adaptations that may enable resistance to, or delay of the onset of desiccation stress, and thus facilitate survival, have received comparatively little attention compared with physiological responses to drying (e.g. [4], [5]).

Recalcitrant seeds exhibit a number of adaptations/traits that reduce the likelihood of desiccation induced mortality. These include: (1) large seed size [3], [6]; (2) occurring most frequently in comparatively aseasonal and moist habitats [7]; (3) being dispersed in the wet-season [2], [3]; and (4) being less likely to be dormant [7] and thus able to germinate relatively rapidly after shedding [2], [3]. Although more unusual, some species with recalcitrant seeds are also known to occur in species from arid and distinctly seasonal habitats, e.g. some South African Amaryllidaceae [8], *Vitellaria paradoxa* (Sapotaceae) from Burkina Faso [6] and certain *Quercus* species [9], [10], [11]. Thus, it is possible that recalcitrant seeds from such environments have adaptations that restrict the rate of water loss compared with congeners from more mesic environments.

Gross seed morphology such as seed size and shape may regulate the rate of water uptake and loss by impacting on the surface area: volume ratio. This has been shown to be the case for the Meliaceae where recalcitrant seeded species tend to have larger, more rounded seeds than species with desiccation tolerant seeds [12]. Slower drying rates have also been reported for recalcitrant seeded species in comparisons with congeners with desiccation tolerant seeds (e.g. *Dipterocarpus* species [13]), although the basis for these drying rate differences is unknown.

With c. 450 species, *Quercus* is the largest and most widely distributed genus in the Fagaceae. Species from the genus are widespread in the Northern Hemisphere, in habitats ranging from temperate and tropical forests to dry thorn scrub and semi-desert [9], [10], [11]. The fruits of *Quercus* species consist of a single seed contained within a hard pericarp (fruit wall) and with one possible exception all those studied to date have been reported to be recalcitrant [14], [15]. Further, for fruits the lack of a relationship between fruit size and drying rates [14] suggests that instead, pericarp anatomical characteristics may be important for regulating the rate of water loss/uptake.

Water uptake and loss by fruits in the genus *Quercus* has been studied previously. For example, a micro-morphological study of *Q. suber* [16] suggested that based on the pericarp having palisade cells and a cuticle (i.e. hypothesized to be impermeable), the scar (the point of attachment of the pericarp to the parent plant), which contains vascular bundles would be the main point of water uptake/loss. However, this study did not investigate the rates of water uptake or loss across the different pericarp components. In addition, a study of water uptake in *Quercus* showed that, for *Q. nuttallii* and *Q. palustris* the scar was the main route for water uptake [17]. Conversely the same study found that for *Q. falcata var. pagodaefolia* and *Q. rubra* water uptake through the scar did not differ significantly from that of the remainder of the pericarp. However, the anatomical basis of these differences in rates of water uptake was not investigated. Consequently, the mechanism(s) regulating water loss and subsequent water uptake (imbibition) in fruits of *Quercus* species are still poorly known.

In this study, we investigated the water uptake and pericarp anatomy of fruits of nine *Quercus* species. Our species selection comprised members of subgenera *Quercus* and *Cyclobalanopsis* which occur in a variety of different vegetation types such as temperate deciduous and subtropical evergreen broad-leaf forests. Our study aimed to (1) understand the route of water uptake through the pericarp and measure the differences in rates of water uptake between the different areas of the pericarp (i.e., the basal detachment scar, the apex with the remnants of perianth and styles and the remaining area between the two); (2) assess how the anatomical properties of the pericarp influence water uptake in *Quercus* fruits and (3) relate rates of water uptake to rates of water loss during drying to assess the relevance of fruit anatomical differences for fruits dispersed into the natural environment and facing the risk of desiccation induced mortality.

## Materials and Methods

### Fruit Lots and Fruit Characteristics

**Figure 1:**
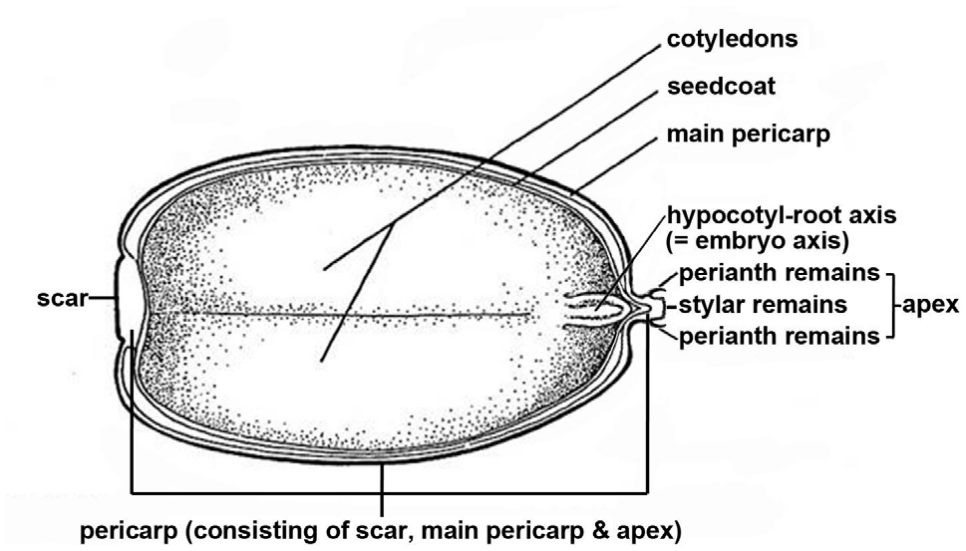
Diagram of a *Quercus* fruit showing the pericarp components, hypocotyle-root axis (embryo axis) and cotyledons. Figure is modified from [31].

Nine *Quercus* species were chosen spanning a range of vegetation types across south and southwest China (Table 1). Fruits (single seeds surrounded by a pericarp; see Figure 1 for details of fruit structure) were collected in late autumn at the time of natural dispersal (Table 1) from a minimum of 5 individual trees per species. Fruits from the individual trees were immediately pooled into a single batch per species. All fruits were collected from land owned by the national government with all appropriate collection permits received from Kunming Institute of Botany, CAS and Guangxi Institute of Botany, CAS. None of the species collected were protected. Upon receipt, fruits were stored at either 15°C (sub-tropical origin) or 5°C (temperate origin; Table 1) as reported previously [14]. Recalcitrant seeds and fruits of many tropical and sub-tropical species are killed when stored at low temperatures (e.g. 5°C [18]). Consequently, 15°C was used for fruits of sub-tropical origin to minimise the likelihood of chilling induced viability loss.

**Table 1:** Collection information and vegetation types in which the 9 *Quercus* species investigated in this study occur.

Species	Collection locationand altitude (m)	Collection date	Habitat type	MAP (mm)	Aridity index‡
***Q. annulata*** Sm.†	Guilin, Guangxi (N 25°01′,E 110°17′), 150 m	Nov 2007	HSEBLF: monsoon evergreen forests	1000–2000	1.3733
***Q. fleuryi*** Hickel & A. Camus†	Guilin, Guangxi (N 25°01′,E 110°17′), 230 m	Nov 2007	SSEBLF: karstic (limestone) landscapes	900–1200	1.0892
***Q. lamellosa*** Sm.#	Jingdong, Yunnan (N 24°43′,E 100°30′), 2,398 m	Oct 2006	Tropical monsoon forests and rain forests	2000–3000	1.5726
***Q. multinervis*** (W. C. Cheng &T. Hong) Govaerts†	Hunan (N 27°25′, E 112°86′), 1,090 m	Oct 2007	HSEBLF: North subzone	1000–2000	1.4986
***Q. schottkyana*** Rehder & E. H. Wilson#	Kunming, Yunnan (N25°01′, E102°41′), 1,900 m	Nov 2007	SSELBF: valleys on the Yunnan plateau, dry-hotvalley, middle mountainous valley	600–1600	0.8501
***Q. sichourensis*** (Hu)C. C. Huang & Y. T. Chang†	Yunnan (N23°45′, E 104°52′), 980 m	Nov 2007	HSEBLF: karstic landscapes	900–1200	1.1298
*Q. fabri* Hance#	Kunming, Yunnan (N 24°97′,E 102°62′), 2,050 m	Oct 2007	HSEBLF: evergreen & deciduous forests,evergreen forests	800–2000	1.2282
*Q. franchetii* Skan#	Kunming, Yunnan (N25°01′, E102°41′), 1,900 m	Oct 2007	SSELBF: valleys on the Yunnan plateau, dry-hotvalley	600–1200	0.513
*Q. variabilis* Blume#	Kunming, Yunnan (N25°01′, E102°41′), 1,900 m	Oct 2007	Warm temperate deciduous broad-leaf forests; HSEBLF: evergreen & deciduous forests,evergreen forests; SSELBF: karstic landscapes	600–2000	1.0977

Data is compiled from [27], [28], [29]. Species names in bold are subgenus *Cyclobalanopsis*, the remaining species are subgenus *Quercus*: *Q. fabri* is from section *Quercus*, *Q. franchetii* and *Q. variabilis* are from section *Cerris*. HSEBLF = humid subtropical evergreen broad-leaf forests; SSEBLF = semi-humid subtropical evergreen broad-leaf forests. MAP = mean annual precipitation.

† stored at 15°C prior to experimentation;

# stored at 5°C prior to experimentation;

‡ from CGIAR-CSI GeoPortal [30]: the aridity index is the ratio of annual precipitation to annual potential evapo-transpiration. Consequently, the smaller the number the more arid the environment.

Fruits were desiccated using silica gel according to the methods described previously [14]. Fruit dry mass was determined by drying 25 individual fruits per species at 103°C for 17 hours which is the standard temperature and duration recommended by the International Seed Testing Association [19]. Germination tests were conducted by incubating a total of 25 fruits (split over two sub-samples of 12 and 13 fruits, respectively) at 15, 20 and 25°C on a 1 cm thick layer of 1% agar in distilled water inside closed plastic boxes (size: 174 × 115 × 60 mm). Fruits of the larger fruited species (*Q. fleuryi* and *Q. sichourensis*) were split over three-subsamples (2 × 8, 1 × 9 fruits), and four subsamples (3 × 6, 1 × 7 fruits), respectively.

Germination was defined as radicle protrusion by at least 2 mm. Germination was recorded weekly with the experiments terminated once no further germination was recorded for at least four consecutive monitoring events. During the weekly germination scoring, the position of the fruits on the agar was changed and the position of the germination boxes within the growth chamber re-randomised.

### Water Uptake by Fruits

For the purpose of this study, the pericarp of *Quercus* fruits was sub-divided into three areas (Figure 1): (1) the apex (marked by the remnants of the perianth and styles [20]); (2) the basal detachment scar where the fruit separates from its cupule; and (3) the remaining area between the apex and the scar which represents the bulk of the surface area of the pericarp and which will hitherto be referred to as the ‘main pericarp’. The area comprising both the apex and the main pericarp (i.e. the pericarp excluding the scar) was referred to as the ‘non-scar area’.

In order to examine the permeability of the different areas of the pericarp to water, intact fruits were either partially or fully coated with dental wax (Cavex Holland BV, the Netherlands) [17]: (1) control (pericarp not covered with wax), (2) negative control to test the impermeability of wax covering (pericarp entirely covered with wax; this was only conducted for fruits of *Q. annulata* and *Q. franchetii*); (3) fruits partially covered with wax so as to seal the non-scar area (main pericarp and apex) (4) fruits partially covered with wax to seal the scar only; (5) fruits partially covered with wax to seal the main pericarp and the scar (i.e. the entire fruit except the apex). Finally, (6) the cuticular wax was removed visually from the pericarp with 95% ethanol [21] (only for fruits of *Q. annulata*, *Q. fleuryi*, *Q. franchetii*, *Q. schottkyana* and *Q. variabilis*). Each treatment was applied to a total of 45 fruits per species (3 replicate tests using 15 each). For *Q. variabilis*, due to severe weevil infestation, only 5 fruits were available for each replicate test of treatments 1, 3, 4, and 5.

Prior to imbibition (rehydration of the fruits), total fruit weight, after sealing, was recorded for each replicate test (W_fw0_). Subsequently, the fruits were placed inside plastic boxes where they were held completely submerged in distilled water by a plastic net cover and kept at 20°C for 24 h [17]. Total fruit weight for each replicate test was determined again after 24 hours of imbibition (W_fw1_). Before weighing, the fruits were blotted dry with blotting paper to remove any free water. After imbibition, the dry mass of all the fruits in each replicate (DM) was determined. For each individual treatment, water uptake was calculated as the increase in moisture content relative to the dry weight of the fruits (MC_Δ_):
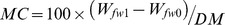
W_fw0_ = weight of the fruits (incl. wax) prior to imbibition; W_fw1_ = weight of the fruits (incl. wax) after imbibition.

### The Surface Area of the Scar, the Main Pericarp and the Apex

Ten randomly chosen fruits of each species were used to determine the mean surface areas of the apex, scar and main pericarp. In order to overcome the problem of the curvature of the fruit surface, the pericarp of each fruit was cut into small pieces and the outlines of the pieces drawn onto graph paper. The total pericarp surface area of a fruit was determined by counting the cells (1 mm^2^ per cell) covered by all the pieces of its pericarp. The area covered by the remnants of the perianth and the styles marking the apex was measured using image analysis software (Axiovision version 3.1., Carl Zeiss AG, Germany) which allowed direct accurate surface measurements in digital photographs taken with a digital microscopy camera (Zeiss Axiocam) mounted on a stereomicroscope (Zeiss Stemi SV11). The mean of these results was used to represent the surface area of the apex (S_0 a_), the scar (S_0 s_), the main pericarp (S_0 mp_), whole pericarp/fruit (S_0__p_) and the non-scar area (S_0 ns_ = S_0 s_ + S_0 mp_) (S_0_ stands for relative surface area of each component in the following equations). The scar surface ratio (SSR) was calculated as the proportion of the scar area relative to the surface area of the whole pericarp. The ratio of the measured surface area of each component to the fruit dry mass of the individual fruit (based on 10 fruits per species) (S_0_/DM_0_) was then scaled to the fruit dry mass of all the fruits in a replicate (S/DM). Therefore, the total surface area of each component of all the fruits in a replicate can be calculated by:
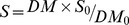



### Observations on the Route of Water Uptake

Whole fruits of each species were submerged in 5‰ Methylene Blue solution for 8 hours [22] and then sectioned transversely (16 µm thick) with a cryostat microtome (model CM 3050 S, Leica Instruments GmbH) across the middle of the fruit. Fruits of *Q. fleuryi* were also sectioned longitudinally (along the transmedian longitudinal axis). For each species, the thickness of the main pericarp area was measured in transverse sections taken across the central part of the fruits of each species using the Stemi SV11 stereomicroscope. On each section, the pericarp thickness was measured in 2 randomly chosen locations to obtain a mean value for pericarp thickness.

Anatomically, the main pericarp area consists of an outer sclerenchymatous palisade layer underlying the cuticle (i.e. a single layer of dense thick-walled palisade cells without any intercellular spaces) and, to the inside, several layers of collapsed, undifferentiated parenchyma penetrated by vascular bundles. Measurements were made on 5 sections per species with the Axiocam and Axiovision 3.1 software. Cuticle thickness was measured using a compound microscope (Nikon OPTIPHOT; 40× magnifications) on 5 sections on which the cuticle had been stained with Sudan.

### Determination of the Area of the Vascular Bundles in the Scar

For each species, the area of the scar (S_s_) and the area of each vascular bundle within the scar (S_vb_) was measured, for 5 individual scars, using the Stemi SV11 with the Axiocam and Axiovision 3.1 software. The vascular bundle ratio VBR, i.e. the proportion of the scar area comprising vascular bundles was calculated:
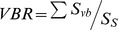



### SEM

Scanning electron microscopy (SEM) was used to examine the microstructure of the surface of the scar and the apex. Scars of the 9 species were mounted on aluminium SEM stubs and sputter-coated (2×3 min, 40 mA) with a platinum-gold alloy, using an EMITECH K550 Sputter Coater (Emitech Ltd., UK). Specimens were examined and photographed using a HITACHI S-4700 Scanning Electron Microscope at an accelerating voltage of 2.0 kV and working distance of 12.0 mm.

### Statistical Analysis

Desiccation curves for the 9 species of moisture content against drying time were analysed by plotting water content to a log_10_ scale and then using the slope of the initial linear part of the drying curve (*k*) as a measure of relative drying rates. The slope of the line was assessed using least squared linear regression. A log_10_ transformation linearises the initial rapid decline in moisture content that is seen when desiccating fruits with this approach having been used previous in studies of recalcitrant fruits [6].

Arcsine transformed moisture content percentages and rates of water uptake were analysed statistically by analysis of variance (One-way ANOVA). Subsequently, the mean values for each treatment were compared using Fisher’s *post hoc* least significant difference test (LSD). The comparison of the accumulated moisture contents by the main pericarp (calculated data) was analysed with a *t*-test. Linear regression was used to test for the effect of the different pericarp attributes on water uptake rates/drying rates. All the tests were performed using MINITAB® 11.21 (Minitab Inc.).

Multiple regression, implemented in Minitab 11.21, was used to assess the relative contribution of the different pericarp components to overall rates of water uptake. Specifically, for the rate of water uptake (g d^-1^), the following were assessed in the regression analysis: the area of the apex (mm^2^); the thickness of the cuticle and palisade layer combined (mm); the thickness of the vascularised inner layer in the main pericarp (mm); the total area of vascular bundles exposed in the scar (mm^2^); and phylogeny (subgenus *Cyclobalanopsis* versus subgenus *Quercus*). The thickness of the cuticle and palisade layer was combined since these two measures were highly correlated (r = 0.869, *df* = 7, *P*<0.001). Initially all terms were included in the model and at each step in the procedure the variable with the smallest (non significant) partial correlation was dropped from the model until only significant (*P*<0.05) terms remained in the model [23].

## Results

### Fruit Viability and Drying Rates

**Figure 2:**
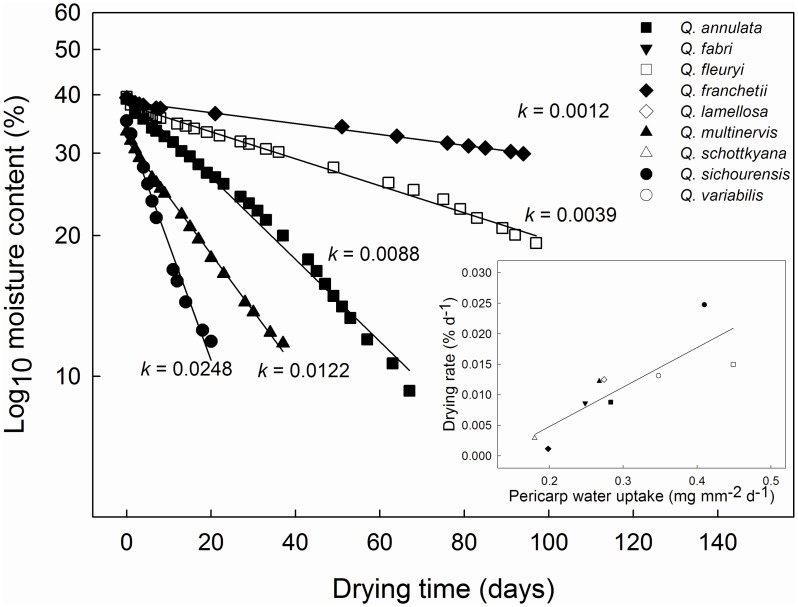
Representative drying curves for fruits of 5 *Quercus* species. The presented curves span the entire range of drying rates observed for the full 9 species. For each species, 37 fruits were desiccated at 15°C using silica gel. The slopes of the fitted lines (*k*) are given on the figure. Inset: the relationship between drying rates (*k*) and rates of water uptake for fresh fruits of the 9 *Quercus* species imbibed in water for 24 h.

The high germination percentages (for fruits sown within 2 weeks of receipt in the laboratory) for most of the species (≥76% for all except *Q. lamellosa*, *Q. multinervis* and *Q. sichourensis*) demonstrated the good quality of the fruits used for this study (Table 2). The whole fruit water content for the 9 species ranged from 33.1–47.4% for *Q. annulata* and *Q. lamellosa*, respectively. Across the study species, the slopes of the drying curves ranged from 0.0012 to 0.0248 for *Q. franchetii* and *Q. sichourensis*, respectively (Table 2; Figure 2).

**Table 2:** Fruit dry mass, viability and drying rates (see also Figure 2) of the 9 *Quercus* species studied here.

Species	Seed dry mass (g)	Maximum germination (%) (temperature)	Drying rate (*k*)
*Q. annulata*	1.89±0.06	76.6 (15°C)	0.0088
*Q. fabri*	0.74±0.03	96.2 (20°C)	0.0086
*Q. fleuryi*	6.41±0.19	96.3 (15°C)	0.0150
*Q. franchetii*	0.59±0.02	100 (15°C)	0.0012
*Q. lamellosa*	4.55±0.30	32.7 (20°C)	0.0125
*Q. multinervis*	1.05±0.05	44.6 (25°C)	0.0122
*Q. schottkyana*	0.59±0.04	95.8 (25°C)	0.0029
*Q. sichourensis*	6.25±0.40	28.0 (25°C)	0.0248
*Q. variabilis*	1.33±0.06	77.6 (20°C)	0.0131

Germination data are means and fruit dry mass data are means ± 1SE.

### Scar and Main Pericarp Anatomy

**Figure 3:**
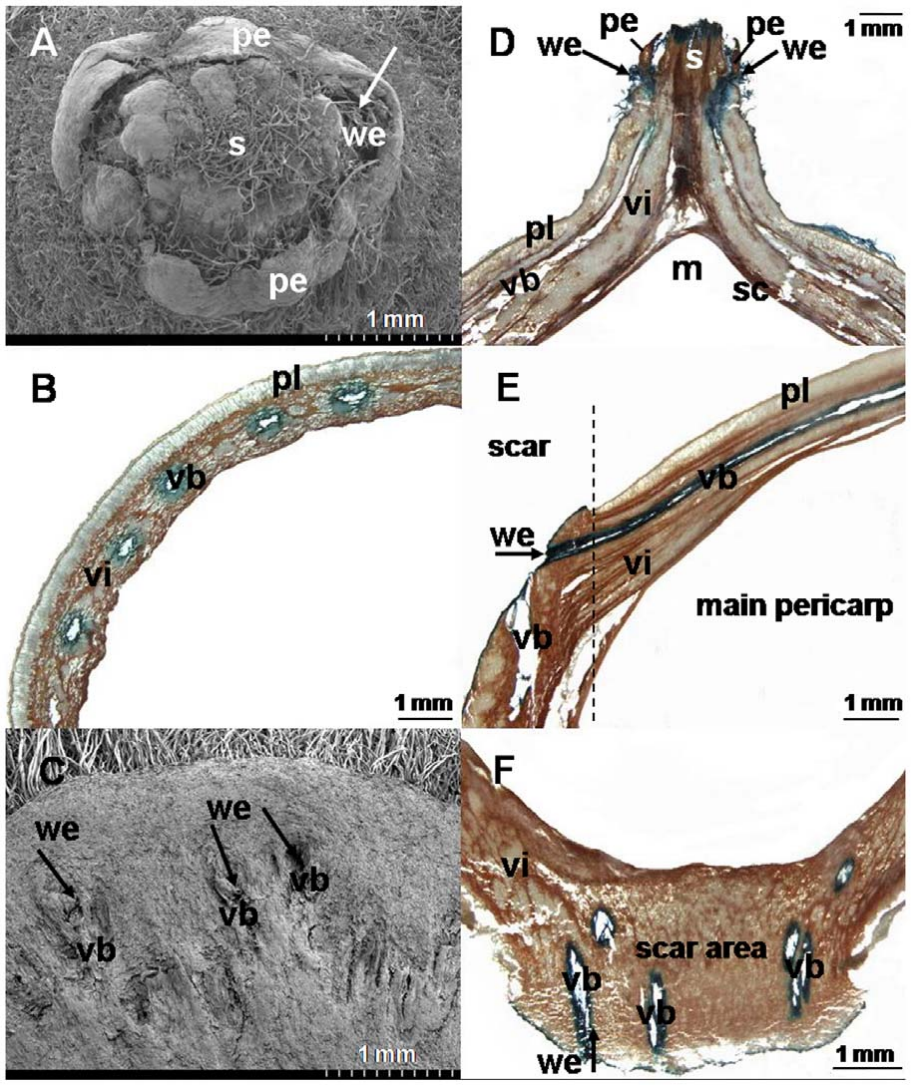
The routes of water flow into fresh *Q. fleuryi* fruits when imbibed in water. (A) SEM photograph of apical end of fruit; (B) cross-section of the main pericarp area of the fruit following imbibition in 5‰ Methylene Blue for 8 h, showing that vascular bundles in vascularised inner pericarp layer stained blue; (C) SEM photograph of the surface of the scar, showing the open ends of the vascular bundles of the pericarpial vascular system; (D) longitudinal section through the apex, with Methylene Blue staining showing that water entered into the fruit at the point where the remains of the perianth segments are attached; (E) longitudinal section of the scar (to the left of the dashed line) and the main pericarp (to the right of the dashed line), with Methylene Blue staining showing that water enters from the scar directly into the pericarpial vascular system that penetrates the inner pericarp layer; (F) longitudinal section of the scar with Methylene Blue staining only the vascular bundles whereas the surrounding water-impermeable sclerenchymatous tissue remains unstained; m = micropylar area of the fruit (embryo missing in photograph); pe = perianth remains; pl = palisade layer; s = stylar remains; sc = seed coat; vb = vascular bundle; vi = vascularised inner pericarp layer; we = point of water entry. Arrows show the points of water entry.

Across the species, the surface area of the scar as a percentage of the area of the entire pericarp (SSR) ranged from 4% (*Q. fabri* and *Q. fleuryi*) to 36.9% (*Q.sichourensis*). Scars of all the species were composed of small and undifferentiated parenchyma cells (about 20–26 μm in diameter) with no cuticle or external layers (Figure 3C). The vascular bundles were arranged symmetrically as a circle in the scar (Figure 3C) although the number (11–67) and size of the vascular bundles (0.02–0.31 mm^2^) differed across the species (Table 3). Further, across the species, the surface area of the vascular bundles as a percentage of the surface area of the entire scar (VBR), ranged from 0.1% (*Q. sichourensis*) to 16% (*Q. fleuryi*) (Table 3).

**Table 3:** Anatomical characteristics of the scar, the main pericarp and the apex of the 9 *Quercus* species studied here.

Species	Scar				Main Pericarp			Apex
	SSR (%)*	S_vb_ (mm^2^)	n_vb_ (n)	VBR (%)*	δ_c_(mm)	δ_pl_ (mm)	δ_vi_ (mm)	S_a_ (mm^2^)
*Q. annulata*	6.3±0.4^ab^	0.17±0.006^a^	27±3^ab^	6.1±0.3^a^	0.013±0.001^ab^	0.24±0.01^a^	0.31±0.02^a^	1.35±0.10^abc^
*Q. fabri*	3.9±0.3^c^	0.02±0.00^b^	29±3^ab^	3.6±0.2 ^b^	0.010±0.001^a^	0.21±0.01^ab^	0.18±0.01^a^	0.88±0.05^bc^
*Q. fleuryi*	4.1±0.1^c^	0.31±0.13^c^	50±8^c^	16.0±1.4^c^	0.063±0.003^c^	0.37±0.02^c^	1.17±0.07^b^	5.83±0.40^d^
*Q. franchetii*	5.8±0.1^b^	0.02±0.01^b^	11±5^d^	1.2±0.6^de^	0.011±0.001^ab^	0.20±0.01^bd^	0.21±0.01^a^	0.71±0.08^b^
*Q. lamellosa*	17.5±0.9^d^	0.14±0.04^a^	67±6^e^	3.4±0.3^b^	0.013±0.001^ab^	0.22±0.02^ab^	1.37±0.03^c^	6.41±0.46^d^
*Q. multinervis*	7.6±0.4^a^	0.04±0.02^b^	47±17^c^	3.8±1.1^b^	0.015±0.001^b^	0.15±0.01^e^	0.16±0.01^a^	1.99±0.18^a^
*Q. schottkyana*	6.3±0.3^ab^	0.03±0.01^b^	34±7^a^	3.4±0.3^b^	0.011±0.001^ab^	0.17±0.01^de^	0.19±0.01^a^	1.51±0.13^ac^
*Q. sichourensis*	36.9±0.6^e^	0.02±0.00^b^	43±11^ac^	0.1±0.02^e^	0.010±0.001^a^	0.19±0.01^b^	1.77±0.13^d^	4.23±0.44^e^
*Q. variabilis*	12.2±0.7^f^	0.07±0.03^b^	23±4^b^	2.1±0.4^bd^	0.014±0.003^ab^	0.15±0.02^e^	0.47±0.05^e^	1.54±0.21^ac^

The scar surface ratio (SSR) and the surface area of the apex (S_a_) were measured on 10 individual fruits; the area of the individual vascular bundle (S_vb_), the number of the vascular bundles in a scar (n_vb_), the vascular ratio of the scar (VBR) on 5 individual scars; and the thickness of the cuticle (δ_c_), palisade layer (δ_pl_), and vascularised inner pericarp layer (δ_vi_) on 5 slices of the main pericarp. Data are means ±1SE. Values with the same letters within each column were not significantly different at p>0.05 (One-Way ANOVA with Fisher’s LSD *post hoc* analysis).

* The ratio presented as a percentage.

**Figure 4:**
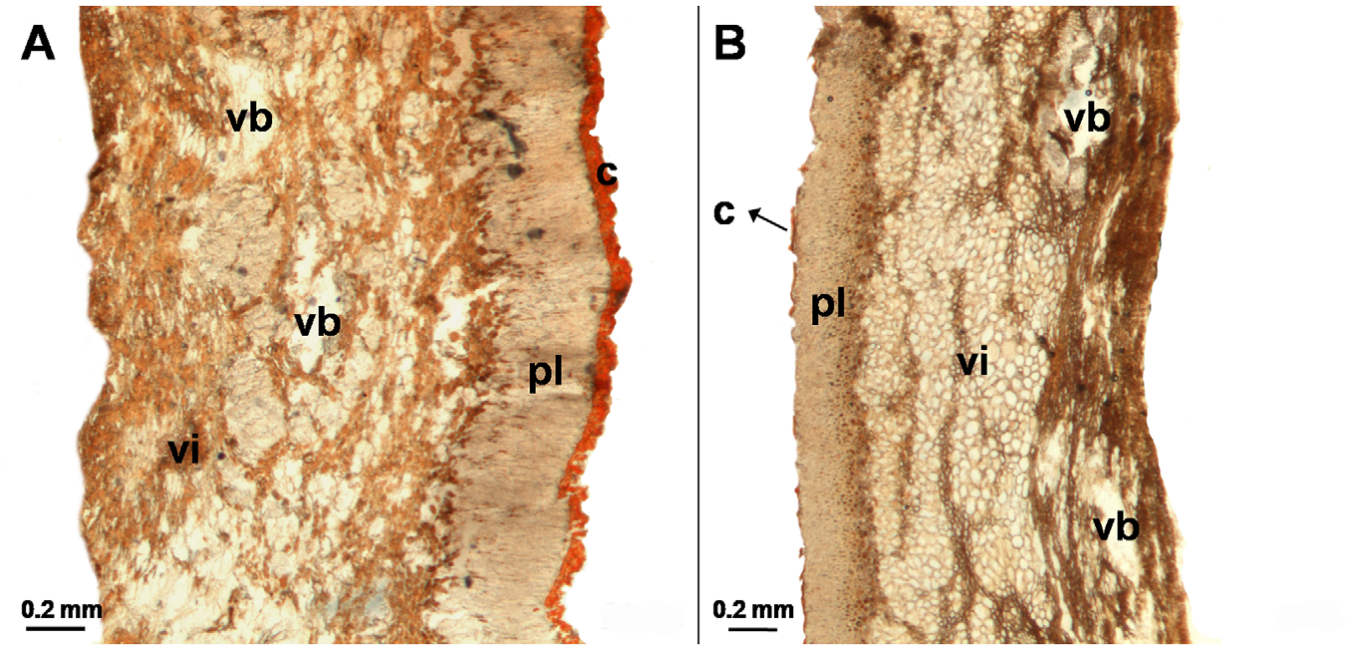
Transverse sections of the main pericarp of *Q. fleuryi* (A) and *Q. lamellosa* (B) stained with Sudan which colours the cuticle red. c = cuticle (stained red); pl = palisade cell layer; vb = vascular bundles; vi = vascularised inner layer.

The main pericarp of the study species was composed of an external cuticle layer (0.010–0.063 mm thick), a palisade layer (0.15–0.37 mm thick) and a parenchymatous layer (0.16–1.77 mm thick, representing 46.8% to 90.4% of the total main pericarp) of largely undifferentiated thin-walled cells penetrated by vascular bundles (hereafter referred to as the vascularised inner layer: Table 3; Figure 4). With the exception of *Q. lamellosa*, which had a discontinuous cuticle layer (Figure 4), the cuticle of the other 8 species covered the main pericarp homogeneously and smoothly.

### Water Uptake Route in *Quercus* Fruits

For whole *Q. fleuryi* fruits which had been imbibed in Methylene Blue solution, the vascular bundles in both the scar and the main pericarp were dyed blue (Figures 3B, E and F). The solution first flowed into the vascular bundles of the scar and then through the vascular bundles into the main pericarp area. At the apex, the solution flowed in from the interface of the remnants of the styles (Figure 3D).

**Figure 5:**
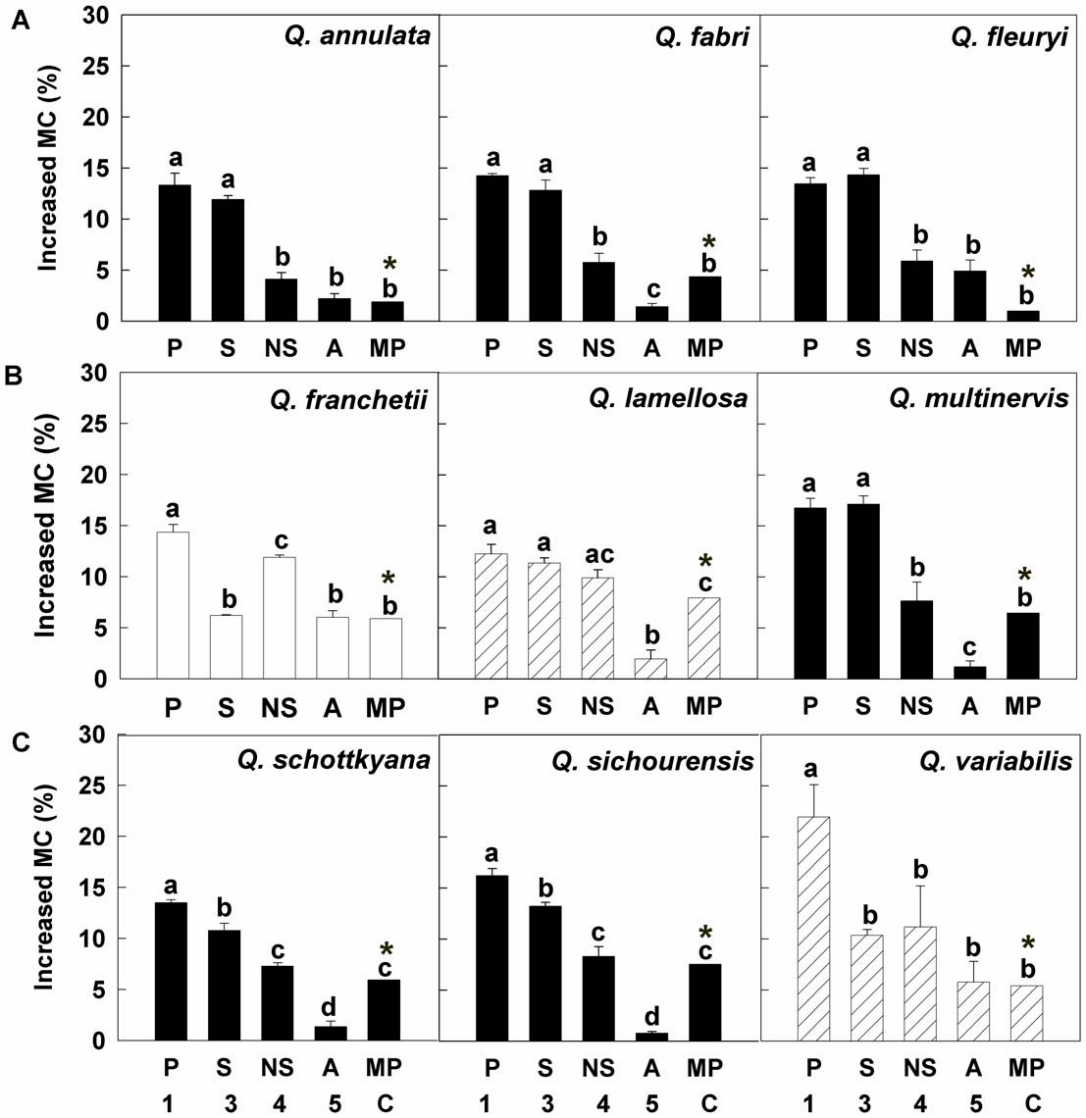
The increase in moisture contents for fruits of the 9 species following imbibition in water for 24 h. The figure shows the moisture content increase of the whole fruit when different components of the pericarp were sealed with wax. Treatments were as follows: no part of the pericarp was sealed with wax (P, treatment 1); the entire pericarp, except for the scar, was sealed with wax (S, treatment 3);the entire pericarp, except for the non-scar area, was sealed with wax (NS, treatment 4); the entire pericarp, except for the apex, was sealed with wax(A, treatment 5). The final treatment (MP, C) represents water uptake through the main pericarp, i.e. assuming the scar and apex were sealed. This value was derived by subtracting the mean of the water uptake through the apex (A, treatment 5) from that through the non-scar area (NS, treatment 4). Bars (mean ±1SE) with the same letters (a-d) are not significantly within each species were not significantly different at p>0.05 (One-Way ANOVA with Fisher’s LSD *post hoc* analysis). The asterisk (*) above the bars showed the increase in moisture contents when only the main pericarp are unsealed (MP, C) were calculated by the mean values without standard errors. Data for the increase in moisture content were analyzed using 1 sample t-test to test for effects of the pericarp sealing treatments on water uptake.

Across species there were differences in the relative contribution of the different pericarp components to the overall water uptake by fruits. Three broad patterns of water uptake could be discerned from the data (Figure 5). For *Q. annulata*, *Q. fabri*, *Q. fleuryi*, *Q. multinervis*, *Q. sichourensis* and *Q. schottkyana*, the scar was the main route of water uptake. For *Q. franchetii*, the non-scar area was the main route of water uptake. Finally, for *Q. lamellosa* and *Q. variabilis*, both the scar and non-scar area had a similar relative role in water uptake.

**Figure 6:**
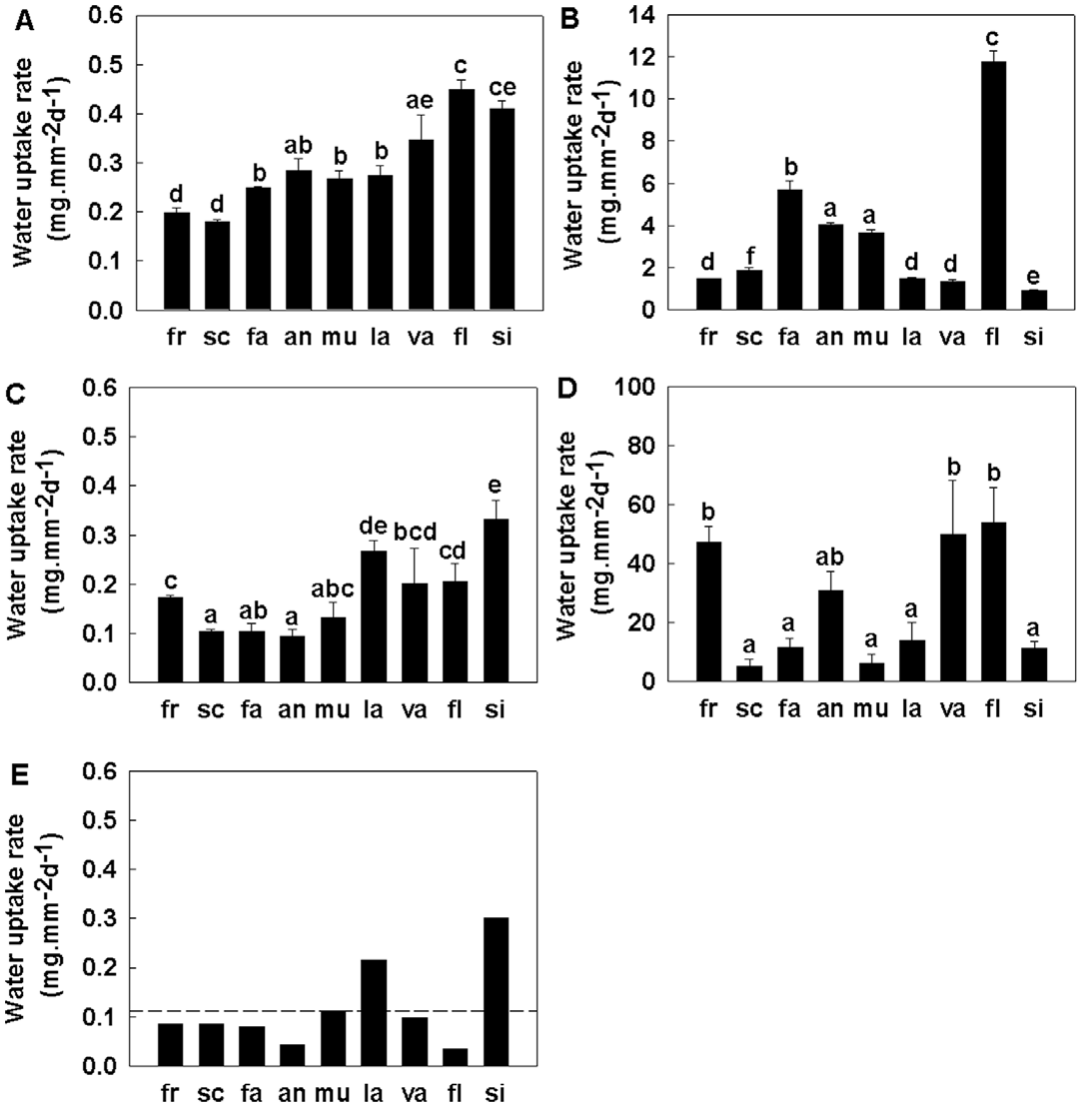
Rates of water uptake through the various pericarp components of 9 *Quercus* species after 24 h imbibition. Fresh fruits were used in this experiment. Figure shows imbibition through the pericarp (A, treatment 1), the scar (B, treatment 3), the non scar area (C, treatment 4), the apex (D, treatment 5), and the main pericarp (E, by calculation); an = *Q.annulata*, fa = *Q. fabri*, fl = *Q. fleuryi*, fr = *Q. franchetii*, la = *Q. lamellosa*, mu = *Q. multinervis*, sc = *Q. schottkyana*, si = *Q. sichourensis*, va = *Q. variabilis.* Bars (mean ±1SE, in plot A, B, C, D) with the same letters (a-f) are not significantly different from each other (*p*≥0.05). Bars in plot E showed the mean rate of water uptake of the main pericarp which is calculated as the mean increase in moisture content relative to the surface area of the main pericarp. Bars are ordered according to increasing drying rates of the species. Note the different scales on the y-axes.

The relative rates of water uptake between the pericarp components also differed between species. Rates of water uptake (on a per unit area basis) in rank order for the different components were the apex (5.33–53.94 mg mm^−2^ d^−1^) > the scar (0.91–11.78 mg mm^−2^ d^−1^) and > the main pericarp (0. 03–0.30 mg mm^−2^ d^−1^) (Figure 6). Across the species, the rate of water uptake for the whole pericarp (including the three components above) ranged from 0.18 to 0.45 mg mm^−2^ d^−1^. While on a per unit area basis the apex had a high rate of water uptake, only a comparatively small proportion of the absolute volume of water imbibed entered through this area (Figure 5). This apparent discrepancy resulted from the small surface area of the apex compared with the total pericarp surface area (the apex was <0.34% [range 0.15 to 0.34%] of the total area of the whole pericarp).

Across the study species, the drying rates were positively related to the rate of water uptake across the pericarp, i.e. from the outside to the inside of the fruit (Linear regression, R^2^ = 0.72, *P*<0.05; Figure 2 inset).

### Determinants of Water Uptake Rates in *Quercus* Fruits

In a multiple regression analysis of the factors potentially impacting on the rate of water uptake, neither the thickness of the combined cuticle and palisade layers, the area of the apex nor the phylogeny of the species were significant (Multiple regression, *P*>0.05). The two terms that remained in the model following backwards elimination were the thickness of the vascularised inner layer of the main pericarp, and the area of exposed vascular bundles in the scar (Multiple regression, *P*<0.05; Table 4). Thus, the rate of water uptake was positively related to the area of exposed vascular bundles in the scar and the thickness of the vascularised inner pericarp layer.

**Table 4:** Final multiple regression analysis model for the effects of area of vascular bundles within the scar and the thickness of the vascularised inner layer of the main pericarp on the rate of water uptake for 9 species of *Quercus.*

Term	d.f.	Coefficient	*t*-value
***Final reduced model***			
Vascular bundle area (mm^2^)	6	1.022	2.51*
Vascularised inner layer thickness (mm)	6	0.07725	2.59*

The initial full model also included the effects of the area of the apex, cuticle thickness and phylogeny (subgenus *Cyclobalanopsis* versus subgenus *Quercus*). However these terms were not significant and were dropped during the backwards elimination process.

* P<0.05.

The absence of an effect of the cuticle in the multiple regression analysis was consistent with the results of the cuticle removal experiment. Thus, with the exception of *Q. franchetti*, removal of the cuticular wax had no impact on the rate of water uptake for any species in comparison with control fruits with an intact cuticle (One-way ANOVA, *df* = 5, *F* = 0.18–2.14, *P*>0.05). For *Q. franchetii*, the effect of cuticular wax removal was significant (One-way ANOVA, *df*  = 5, *F* = 11.44, *P*<0.05) although wax removal only had a minor effect (water uptake only increased by 0.02 mg mm^−2^ pericarp in 24 h when the cuticle was removed).

## Discussion

### Anatomical Control of Water Flow in *Quercus* Fruits

The vascular bundles in the pericarp of the *Quercus* species studied here comprise a continuous system extending from the scar, throughout the main pericarp to the apex. The scar was the major area where the ends of the vascular bundles of the pericarp were exposed and water flowed directly into the vascular system of the pericarp from the scar. In addition, water could also enter across the main pericarp through the palisade layer below the cuticle and through the vascular bundles within the percarpial vascular system connecting the perianth remains and the scar.In addition, this study showed three patterns of water uptake of the pericarp by *Quercus* fruits. In agreement with previous imbibition work on *Q. nuttallii* and *Q. palustris* from section *Lobatae* [17], and a morphological study on *Q. suber* from section *Cerris* [16], the scar was the major route of water flow into and out of the fruit for most species and for species from both subgenus *Cyclobalanopsis* and section *Quercus* in subgenus *Quercus* (pattern 1). However, for *Q. lamellosa* from subgenus *Cyclobalanopsis*, *Q. franchetii* and *Q. variabilis* from section *Cerris* in subgenus *Quercus*, greater or equal amounts of water flowed through the non-scar area (pattern 2 and 3). This was also reported previously for *Q. falcata* var. *pagodaefolia* and *Q. rubra* from section *Lobatae* [17]. Therefore, the intrageneric differences between the various modes of water uptake through the pericarp do not seem to follow phylogenetic relationships. A lack of a phylogenetic signal was also evident from the lack of a significant effect of phylogeny in the multiple regression analysis of factors affecting the rate of water uptake.

Rates of water uptake across the different pericarp components have been investigated previously [17]. However, this current study is the first to link the anatomical structure and water conducting properties of the pericarp in *Quercus* fruits with actual rates of water uptake and loss. The anatomical characteristics of the respective pericarp components controlled their rate of water uptake. The rate of water uptake through the scar varied between different species and was determined by the total surface area of exposed vascular bundles in the scar, i.e. the product of the density of the vascular bundles in the scar (VBR) and the total area of the scar (both of which varied widely across species). The rate of water uptake through the scar was generally greater than that through the remainder of the pericarp. Hence, the scar is the main passage for water uptake in most species, despite representing just 4% to 37% of the total surface area of the pericarp.

The cuticular wax and the palisade layer of the pericarp have been previously proposed to be barriers to water flow (water uptake and water loss) for *Quercus* fruits [16], [17]. However, their role in regulating the rate of water flow across the pericarp has not tested directly before. In our current study, neither the presence of cuticular wax nor the thickness of the cuticle or palisade layer was related to the rate of water uptake through the main pericarp. Instead, the thickness of the vascularised inner layer was positively related to the rate of water uptake through the main pericarp surface. This suggests that the barrier to water flow across the main pericarp is the permeability of this vascularised inner layer with a thicker layer potentially having more vascular tissue and hence greater rates of water flow.

The stylar end of the fruit is, in addition to the scar, a potential opening through which water can pass easily in *Quercus* fruits. Indeed, this area has previously been suggested to be a water gap in *Quercus* fruits [17], [24]. Our present results show that water entry at the apex occurs where the perianth is attached. While, on a per unit area basis, we found that this region had a high rate of water uptake, it was relatively unimportant in determining overall water uptake into *Quercus* fruits since the area of this channel represented just 0.15 to 0.34% of the total pericarp surface area.

### The Effects of the Pericarp Anatomy on Desiccation and Germination

Rates of water loss (desiccation) and water uptake were related in this study. Thus, these two processes are likely impacted by the same anatomical features of the pericarp in *Quercus*. For some of the *Quercus* species studied here, extremely long drying times were observed. For example, fruits of *Q. schottkyana* were dried with silica gel for 134 d before reaching a moisture content of 14%. For fruits of *Q. franchetii*, it has previously been reported that during drying in silica gel for 164 d the moisture content only decreased by 17% reaching a value of 23% [14]. For these two species, a comparatively small scar (ca. 6% of the pericarp area) with few vascular bundles (ca. 1–3% of the scar area) appears to be the mechanistic basis of limited water loss through the pericarp. In a previous study, slower drying of recalcitrant seeds compared with con-generic species with desiccation tolerant seeds was reported in the family Dipterocarpaceae [13] suggesting adaptation to reduce the likelihood of post dispersal desiccation induced mortality in the desiccation-sensitive seeds.

For most *Quercus* species, the hypocotyl-root axis (embryonic axis, Figure 1) sits at the micropylar region underneath the stylar remains of the pericarp. The axis represents the growing points for germination and in that sense is the most critical part of the fruit: death of the axis from desiccation would prevent germination. The axis of *Quercus* species usually has a higher moisture content at dispersal, compared with the cotyledons and the pericarp, and dries later during desiccation [14]. Our data demonstrating the scar to be a key route for water flow into and out of the fruits, provides a mechanistic basis for the slower drying of the axis, which may maximise axis survival in the event of post dispersal dry spells. It has been suggested previously that during drying, the axis in recalcitrant fruits (and seeds) remains at higher water contents than the remainder of the fruit tissues [25]. However, the location of the axis in relation to routes of water loss/uptake has not been investigated in other species with recalcitrant fruits/seeds and warrants further study.

Low drying rates and their corollary low rates of water uptake, represent a potential trade-off between resistance to drying (and survival during dry period post dispersal) and rapid water uptake and germination. Indeed rapid germination is an adaptation observed in many species with recalcitrant fruits [2], [3] and minimises the likelihood of desiccation induced mortality as well as reducing the window of opportunity for seed predation. However, the slow rates of water uptake and loss observed for some of the *Quercus* species in this study potentially constrain the rate at which germination can occur, increasing the window of opportunity for both desiccation and predation. The two species with the slowest rates of water loss were *Q. franchetii* (subgenus *Quercus*) and *Q. schottkyana* (subgenus *Cyclobalanopsis*). For their respective subgenera, fruits of these two species had the longest germination times at 20°C (mean time to germination = 52 and 83–119 d, respectively) observed for the 9 study species (data not shown). However, more studies are needed to test the effect of germination times on these fruits and their seedling survival in the field.

The Asian monsoon system which mainly shapes the Chinese climate, causes distinct wet and dry seasons across the ranges of the *Quercus* species investigated here. These species usually disperse late in the wet season or the beginning of the dry season. Among these species, *Q. schottkyana* and *Q. franchetii* are particularly widely distributed in semi-humid evergreen forests in southwest China (Table 1) and also occur in dry-hot valleys (similar to savannah environments). In all these environments, fruits of these two species are dispersed just prior to a 6 month dry spell (total precipitation is c. 116 and <99 mm respectively), with germination unlikely to occur until the onset of the next rainy season. This is unusual for species with recalcitrant fruits which are typically dispersed at the start of wet spells [2], [7]. However, it is of note that these two species had the lowest drying rates (and rates of water uptake), associated with small scars of few vascular bundles as well as a thinner vascularised inner layer. These traits combined may enable fruit survival until the onset of rains. In contrast, *Q. lamellosa* and *Q. sichourensis* (both from wetter regions) had a vascularised inner layer 7–9 times as thick. Many *Quercus* fruits are buried (cached) by rodents [26] and it is possible that burial in conjunction with the slow rates of water loss enables fruit survival over extended dry periods. The generality of this association between anatomical traits and the low rates of fruit water loss could be tested using *Quercus* species from dry environments in other parts of the world, e.g. Southern USA or Mexico.

In conclusion, differences in the rates of water flow of the 9 *Quercus* species were related to (1) the relative surface area of the scar; (2) the density of vascular bundles in the scar; and (3) the thickness of the vascularised inner layer of the pericarp. Across species, all three of these attributes varied widely which, coupled with the relationship between water uptake and drying rates, provides a mechanistic explanation for the wide variation in fruit drying rates for these species. These drying rate differences might also impact on fruit survival *post* dispersal in the natural environment although this requires further investigation.
